# A paradigm shift in translational psychiatry through rodent neuroethology

**DOI:** 10.1038/s41380-022-01913-z

**Published:** 2023-01-12

**Authors:** Yair Shemesh, Alon Chen

**Affiliations:** 1 Department of Brain Sciences, Weizmann Institute of Science, Rehovot, 7610001 Israel; 2 Department of Molecular Neuroscience, Weizmann Institute of Science, Rehovot, 7610001 Israel; 3 Department of Stress Neurobiology and Neurogenetics, Max Planck Institute of Psychiatry, 80804 Munich, Germany

**Keywords:** Neuroscience, Psychiatric disorders

## Abstract

Mental disorders are a significant cause of disability worldwide. They profoundly affect individuals’ well-being and impose a substantial financial burden on societies and governments. However, despite decades of extensive research, the effectiveness of current therapeutics for mental disorders is often not satisfactory or well tolerated by the patient. Moreover, most novel therapeutic candidates fail in clinical testing during the most expensive phases (II and III), which results in the withdrawal of pharma companies from investing in the field. It also brings into question the effectiveness of using animal models in preclinical studies to discover new therapeutic agents and predict their potential for treating mental illnesses in humans. Here, we focus on rodents as animal models and propose that they are essential for preclinical investigations of candidate therapeutic agents’ mechanisms of action and for testing their safety and efficiency. Nevertheless, we argue that there is a need for a paradigm shift in the methodologies used to measure animal behavior in laboratory settings. Specifically, behavioral readouts obtained from short, highly controlled tests in impoverished environments and social contexts as proxies for complex human behavioral disorders might be of limited face validity. Conversely, animal models that are monitored in more naturalistic environments over long periods display complex and ethologically relevant behaviors that reflect evolutionarily conserved endophenotypes of translational value. We present how semi-natural setups in which groups of mice are individually tagged, and video recorded continuously can be attainable and affordable. Moreover, novel open-source machine-learning techniques for pose estimation enable continuous and automatic tracking of individual body parts in groups of rodents over long periods. The trajectories of each individual animal can further be subjected to supervised machine learning algorithms for automatic detection of specific behaviors (e.g., chasing, biting, or fleeing) or unsupervised automatic detection of behavioral motifs (e.g., stereotypical movements that might be harder to name or label manually). Compared to studies of animals in the wild, semi-natural environments are more compatible with neural and genetic manipulation techniques. As such, they can be used to study the neurobiological mechanisms underlying naturalistic behavior. Hence, we suggest that such a paradigm possesses the best out of classical ethology and the reductive behaviorist approach and may provide a breakthrough in discovering new efficient therapies for mental illnesses.

## Introduction

Mental illnesses are common in all societies and cultures. In western countries, 10–20% of the population suffers from mental illnesses, including depression, different forms of anxiety, schizophrenia, eating disorders, and addiction [1, 2]. In addition, some developmental brain disorders such as autism spectrum disorder (ASD) or attention deficit and hyperactivity disorder (ADHD) have become increasingly prevalent due to either increased public awareness, modified diagnostics, or lifestyle changes [3, 4]. Remarkably, in many societies during the 20th century, public awareness and the public’s attitude towards mental illnesses have been transformed. For instance, attributing post-traumatic stress disorder (PTSD) to weak willpower or shameful character is less widespread than it was before. Since the end of the Vietnam war, governments have taken medical and economic steps to assist veterans suffering from PTSD [5]. The increased awareness of mental illness has raised the expectation for discovering effective therapeutics, which, unfortunately, has not yet been met [6, 7].

**Fig. 1:**
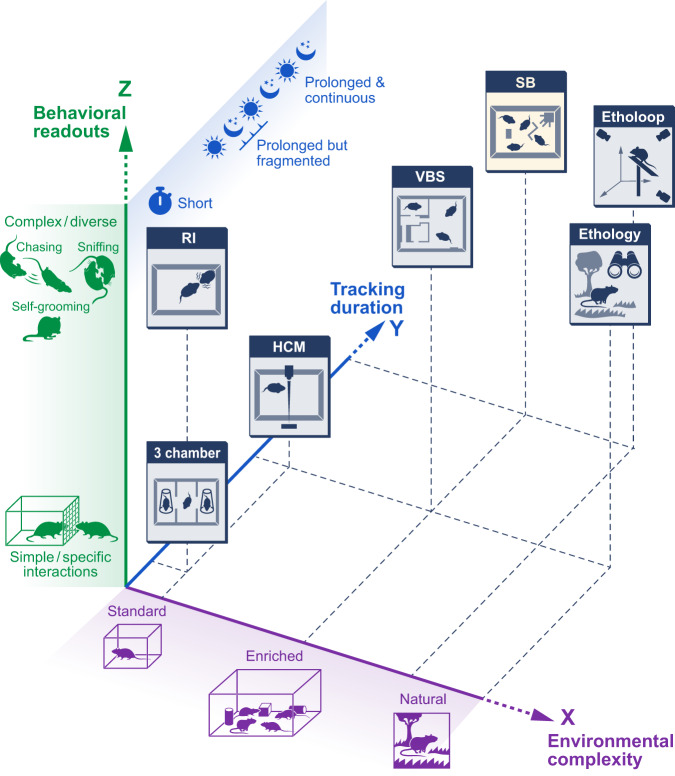
Measuring behavior in animal models. *X axis:* The environmental complexity during behavioral monitoring ranges from a standard and highly controlled setups (e.g., an empty box) to natural. *Y axis:* The duration of behavioral monitoring can last from a few minutes (e.g., 10 min in the 3 chamber social interaction tests) to days or even weeks. Rodents that are kept for prolonged time in a given setup, can either be tracked continually or data can be sampled based on timepoints of interest. *Z axis:* The complexity of the detected behavioral readouts ranges from simple and artificial (e.g., interaction through a mesh with a restricted conspecific) to complex and more ethologically relevant behaviors (e.g., attacks and chases). Various behavioral paradigms can be qualitatively positioned in this three-dimensional space. The relative advantages and drawbacks of the different paradigms/approaches in terms of reproducibly, accuracy and cost effectiveness are discussed in the text. (RI- Resident Intruder Test, HCM- Home Cage Monitoring, VBS- Visible Burrow System, SB- Social Box).

The stagnation in developing remedies for mental illnesses seems puzzling in light of the outstanding technological advances that have been made in brain research over the last three decades, which have deepened our understanding of brain function at different biological levels. Human brain neuroimaging techniques provide insights into functional brain connectivity [8]. Fast genetic screening tools such as genome-wide association studies (GWAS) generate candidate genes to develop hypothesis-driven testing. In addition, manipulation and recording of cell and region-specific neuronal circuits in animal models [9] were anticipated to complement human research and yield a causative understanding of brain disorders’ mechanisms. Such cutting-edge techniques revolutionized behavioral neuroscience research. However, for translational psychiatry, the gap between bench and bedside has remained as wide and deep as a few decades ago [10]. There is a general understanding that this gap is partly due to a limited relevance of the behavioral assays in animals to the human disease, which results in translational loss from humans to animal models and back to humans [11, 12]. Hence, it is time to reevaluate current translational methodologies (Fig. 1) and adopt initiatives that can push the field forward [13–16].

In psychiatry, as opposed to other medical fields, diagnosis is not based on objective biological markers but instead on a categorization of observed and self-reported symptoms [17]. Such a classification system generates considerable overlap between categories. For instance, “social withdrawal” is a devastating symptom typical of ASD, schizophrenia, PTSD, various types of anxiety, major depression, and more. Symptom-based categorization also generates much heterogeneity within each category [15, 18]. For example, two diagnosed patients can display non-overlapping and sometimes even opposing phenotypes for a major anxiety disorder [12]. This limitation in biologically-based diagnostic tools in psychiatry results from the complexity of the diseases. Most mental illnesses are poly-genetic, meaning that what determines the phenotype are many genes that each contribute minor effects and only together become substantial.

Moreover, the relative contribution of each of those genes can vary tremendously between patients in a given syndrome. On top of that, genetic predisposition interacts with environmental factors, such as various past and present stressors, significantly contributing to the complex phenotype of a disorder via epigenetic mechanisms. These diagnosis-related issues of comorbidity, heterogeneity and the multifactorial architecture of the disorders defined in humans make modeling mental disorders in animals extremely arduous since no single animal model can encompass the complexity of a human disorder [19]. Nevertheless, mental illnesses are still disorders of brain circuits, conserved to some extent between mammals and regulating defined behavioral endophenotypes, namely heritable and measurable components of a phenotype that are an intermediate between genes and disease. For instance, fear, social avoidance, reward-seeking, and aggression are endophenotypes that reflect the evolutionarily conserved emotional states between mammals [20–22]. Of note, it is argued that the term ‘emotion’, when used for animal models, does not include the subjective feelings that are based on self-report in humans [23, 24]. Nevertheless, measurable behavioral readouts can still reflect mammals’ common ‘emotional’ state. For example, while it is not feasible to measure sadness as a symptom of depression in mice, it is possible to measure and reveal the mechanisms underlying anhedonia of a rewarding food, avoidance of prosocial stimuli or sexual interactions, and lack of general motivation. In other words, it is possible to measure and characterize the ‘emotional’ state of which ‘sadness’ is a related subjective feeling [20].

It is assumed that conserved primitive emotional states also exist in non-mammalian animals such as flies and worms. In C. elegans, for example, pheromones mediate coping with environmental challenges and promote avoidance of dangers or attraction to resources [25]. Thus, revealing the underlying mechanisms of stress coping in animals with a well-defined nervous system, such as C. elegans, can shed light on the evolutionary processes that led to the complex emotional repertoire of mammals [26]. However, a discussion on this topic is beyond the scope of this review. Here, we promote the view that the quest for improved translational research in psychiatry will benefit from ethologically relevant measurements of complex behaviors in rodents in semi-natural paradigms [27]. We will further discuss the necessity to combine automatic behavioral modeling and analysis in such paradigms, innovative approaches to manipulate the brain and the environment, and translational relevant framework from mice to humans.

## A roadmap to improved translational research

### Research domain criteria

The primary unmet objective in basic psychiatric research is to translate biological measures and findings in the laboratory to the symptom-based categorization as defined in the existing disease-diagnostic systems. The Research Domain Criteria (RDoC) initiative addresses this challenge with a framework that provides a taxonomy for mental illnesses that is based on genetics, behavioral neuroscience, and psychological measures (e.g., self-report in humans) [28]. RDoC is not meant to serve as a diagnostic guide but offers a biological framework for understanding mental health as defined in the existing diagnostic systems. Currently, there are six suggested module domains in RDoC: (1) negative valence (2) positive valence (3) cognitive systems (4) systems for social processes (5) arousal and regulatory systems (6) sensorimotor systems [29]. Each domain contains several constructs that can be considered as endophenotypes. For instance, fear is an endophenotype in the negative valence domain [30]. Each endophenotype can be studied at different biological levels (e.g., genes, molecules, cells, circuits, behavior, and self-report) within a defined developmental and environmental context. RDoC can be visualized as a matrix where the rows describe endophenotypes, and the columns describe biological levels. The matrix is a continuous multi-dimensional description of normal and pathological conditions under various environmental and developmental states.

### Acknowledging the limitations of short reductive behavioral tests

RDoC is a promising framework for experimenting with humans as well as with animal models. For instance, a matrix designed for depression in humans can further serve as a reference for hypothesis testing of various mechanisms and endophenotypes underlying depression in animal models [30, 31]. However, since behavior is the final output of the nervous system, even within the RDoC framework, the quality of the behavioral measures is of cardinal importance. Alas, in animal models, the behavioral building blocks are typically biased towards a reductionist approach, gathered from a battery of strictly controlled, oversimplified, and short paradigms, aiming to maximize standardization and minimize variance by fixing a wide variety of ‘unwanted’ factors [32–35]. On top of that, the tests are usually performed using a single mouse or restricted dyadic interactions outside of the animal’s living environment [32].

Nevertheless, reductionism does not come without a cost [36]; over-simplified paradigms may lead to misinterpretations, anthropomorphic leaps, and eventually questionable face validity (i.e., the similarity in biology between the measured behavior and the human disease). In other words, testing for a specific readout in rodents in a reductionist manner reminiscent of a human illness, outside of holistic ethological context, might depict merely a ‘phenocopy’ of the behavior (same phenotype but different causes). For instance, consider the Forced Swim Test (FST), [37] a test for depressive-like behavior in mice and rats. The animal is forced into a small tank filled with water, and the time it spends swimming and floating without swimming is measured. Floating is interpreted as a lack of motivation or despair and thus a ‘depression-like’ phenotype. However, floating during repeated trials in an inescapable small tank does not necessarily correspond to a lack of motivation. It might instead reflect an adaptive coping strategy or amended learning that swimming is entirely futile, and rescue is on the way. Therefore, floating in the FST might be a phenocopy of the passivity that characterized depression in humans [12]. Of note, the readout in this test is affected by antidepressants. Thus, the FST might exhibit some extent of predictive validity (i.e., how well the measured behavioral readout can predict currently unknown aspects of the disease in humans). Nevertheless, with questionable face validity, it might well be that the antidepressant effect is not mediated by brain circuits specifically relevant to depression [37]. In addition, it is not clear how efficient the test is at detecting novel components with unknown mechanisms of action that might be of therapeutic value [15].

Standardization in the classical behavioral tests is one of their significant advantages; however, it should be acknowledged that many parameters cannot be standardized. Thus, variance is inevitable both within experiments and laboratories [38, 39]. For instance, the number of cage mates in the home cage, which affects the group’s hierarchical structure, varies according to the number of siblings in the original family and mostly is not standardized. In addition, the animal’s social status in its home-cage is not monitored and standardized, though it affects every aspect of the animal’s mental state and behavior. In classical tests such as the FST, some or all of the cage-mates are tested regardless of their social status, which might affect the performance in the test. Also, their social interactions (either aversive or supportive) just before the tests or social buffering of stress after the test are usually not monitored [36, 40, 41].

Other factors that inevitably counteract standardization and reproducibility are the separation and reunion of group members following each test, which increases agonistic behavior between males, also the order and the number of tests, their circadian time, and the ‘experimenter effect’ (gender, handling differences, etc.) [42]. For example, a recent study found that the antidepressant effects of ketamine in mice was only apparent when administered by male experimenters, and established that this was mediated by CRF activation by male experimenter scent [43]. Moreover, from an ethological perspective, focusing on specific readouts in short paradigms might leave relevant behavioral phenomena affected by temporal dynamics in a group context and the underlying neurobiological processes indiscernible [44].

### Modeling endophenotypes of mental illnesses in animals as opposed to ‘animal models’ of mental illnesses

**Fig. 2:**
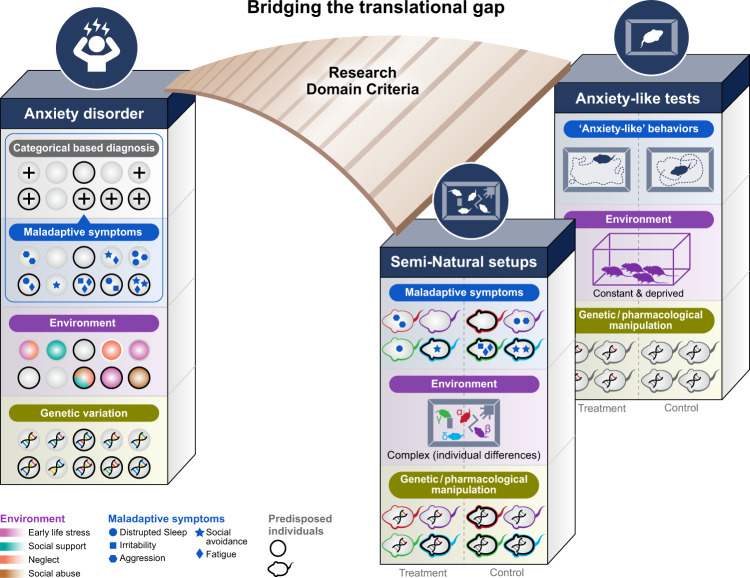
Bridging the translational gap. Translational research of mental disorders using animal models fail to fulfill the expectation to discover novel treatments. Mental illnesses in humans (e.g., anxiety disorders) are complex and influenced by genetic predispositions, past and present environments and the interactions between them. The diagnosis of illness is based on categorized behavioral criterions (‘+’ symbols). On the other hand, anxiety in mice is modeled using short tests that detect ‘anxiety-like’ phenotypes. Though highly controlled, this behavioristic approach is not enough to translate to the complexity of human mental illnesses. Automatic monitoring of complex behavior in groups of mice (semi-natural setup) can reveal individual coping strategies. Combining anxiety-like tests and semi-natural setups while researching endophenotypes that are common across mammalian species have the potential to bridge the gap.

No single genetic or environmentally induced animal model can fully encompass the complexity of human mental illnesses such as ASD or PTSD. Also, no single animal behavioral test can encompass the full behavioral symptoms of human mental illness [45]. However, these translational gaps can be mitigated by an approach that studies mechanisms underlying emotionally related endophenotypes in rodents living in semi-natural setups (Fig. 2). Such an approach uses the best of ethology and behaviorism- the two disciplines developed during the 20^th^ century to decipher the architecture and rules that govern animal behavior. Ethologists study behavior in the animals’ natural habitat and emphasize the importance of innate mechanisms that, together with environmental constraints, shape proper behavioral displays in the face of danger or opportunity. Behaviorists study behavior in controlled laboratory conditions in specific animal models, mainly rodents, and emphasize the importance of learning and conditioning (for example, fear conditioning) as a strategy for coping with the reencounter of a challenge [46]. To illustrate the adaptive value of bringing ethology to the laboratory, we will discuss the validity and drawbacks of some of the canonical classical tests for fear, anxiety, and depression and suggest an ethologically relevant paradigm to study them in semi-natural setups.

#### Classical tests for fear, anxiety, and depression

Fear-conditioning is probably the most extensively studied paradigm in behaviorism [47–49]. Classical Pavlovian fear conditioning is based on associative learning - a biological neutral signal such as a tone (the conditioned stimulus- CS) is coupled to an aversive stimulus such as a mild foot shock (the unconditioned stimulus- US). As a result, the neutral stimulus by itself can elicit a behavioral response (freezing). The associative learning enables the animal to properly respond when re-exposed to the CS alone and avoid the danger, namely the US. Though the subjective component of fear as an emotion might be hard to model in mice, fear conditioning is a behavioral and physiological response to a perceived threat, a response common to all mammals and many other animals [24, 50]. While fear is considered a response to an immediate clear threat [51], anxiety is a sustained response to potential non-immediate danger [52]. Behavioral tests for anxiety in rodents are mostly based on a conflict between exploration, which reflects a tendency to search for resources, and avoidance, which reflects a tendency to minimize exposure to dangers. For instance, in the Open Field Test, the animal is placed for 10 min in an empty, inescapable arena lit by a bright light. The relative time it spends close to the walls, as opposed to in the center of the arena, can be interpreted as a measure of its anxiety levels. In the Light/Dark test, the animal chooses between exploring a bright area versus staying in a dark ‘protected’ chamber. In the Elevated Plus-maze, the animal explores two exposed elevated arms or two walled elevated arms [51]. These tests proved to be insightful in many studies; nevertheless, three significant drawbacks question their ethological validity. First, it is unclear to what extent a given behavior in a test is extreme enough to be considered maladaptive – a behavior that reduces the fitness of the animal – so that it can be classified as anxiety, as opposed to adaptive vigilance [53, 54]. Second, anxiety is a sustained emotional state with temporal dynamics and continuous stressor load, yet these tests last only a few minutes, so at best capture a narrow time window. Third, rodents, like humans, are social creatures, and most anxiety disorders in humans have a strong social component, hence, the lack of social context in the canonical anxiety tests may confound the results [33].

Depression is an affective mood disorder characterized by persistent sadness and lack of energy. Selective serotonin reuptake inhibitors (SSRIs) are the most prescribed antidepressant drugs. However, SSRI responses significantly vary between individuals and are currently evaluated mainly by trial and error [55, 56]. Inducing depression in rodents includes paradigms such as repeated restraint stress, chronic social defeat, or learned helplessness, in which animals are repeatedly exposed to inescapable electric shock, which makes them increasingly helpless until they eventually stop trying to avoid even escapable shocks. Tests for depression in rodents include ‘despair’ paradigms such as the FST (mentioned earlier) or the Tail Suspension Test (TST) [12, 57, 58]. In the TST, a mouse is under the inescapable stress of being suspended by the tail, and the time it spends trying to escape is measured and interpreted as motivation. Performance in both tests is improved by antidepressants and thus has some degree of predictive validity. However, as mentioned for the FST, reduced movement in the TST might be merely a phenocopy of reduced motivation in humans, namely, a similar phenotype mediated by different causes and mechanisms. This possibility is supported by the evidence that the kinetics of the SSRI response differ between human and rodent models of depression. In humans, it takes several weeks for antidepressants to act, whereas their antidepressant effect in rodents is immediate. Moreover, studies that show an improvement of performance in FST or TST in wild-type (“normal”) mice following treatment with SSRI cannot be considered as a predictive validation of these tests to detect mice’s depression-like phenotype [12].

#### Ethologically relevant approach for fear, anxiety, and depression

It has been suggested that the definitions as mentioned above of fear and anxiety might lack ethological relevance [59]. Perusini and Fanselow argue that the current distinctions between fear and anxiety fail to differentiate the two states in terms of their cause (for instance, specific phobia, which is an anxiety disorder, is a response to immediate stimuli) and behavioral consequences (for instance, freezing and hypervigilance can be attributed to both fear and anxiety). They propose the Predatory Imminence Theory (PIT) as a rigorous and comprehensive model of defensive behavior in animals, which can better translate to fear, anxiety, and panic in humans [59]. PIT assumes that the prey’s perception of the predator’s likelihood of being preyed on determines the defensive behavioral response. In other words, the imminent threat and its severeness determine a behavioral response that can be divided into three modes: pre-encounter, post-encounter, and circa-strike. Pre-encounter involves higher vigilance and risk assessment when leaving the nesting area to forage for food. When such behavior is displayed in a non-adaptive manner, namely, when it does not maximize the probability of getting food while avoiding predation, it reflects anxiety. Post-encounter behaviors involve freezing to decrease detection by the predator. This behavior should be displayed when there are indications that a predator is present. If no predator is present, it resembles fear. Circa-strike behavior, which includes jumping and biting the attacker, should be displayed when contact with the predator is already inevitable. If displayed in other contexts, it resembles panic. Implementing PIT in a semi-natural laboratory setup by modeling realistic dangers and testing for innate responses can lead to new insights into the mechanisms underlying maladaptive responses to threats [60, 61]. Predator odors, sounds, or visual stimuli were used previously in the laboratory and can be incorporated into semi-natural paradigms. However, it should be kept in mind that repeated exposure to a threat that is not followed by a real danger will rapidly lead to habituation. Thus, careful design and interpretation are needed. Notably, attention to the design of shelters in a semi-natural setup enables continued exposure to threats from an aggressive conspecific in a way that prevents both habituation and injuries. In such a setup, displays of fleeing, avoidance, submission, risk assessment, and decision-making (for instance, approaching or avoiding a more rewarding food) can be quantified continuously and automatically over days. Moreover, the consequences of maladaptive decisions, in terms of losing resources such as mates, can be monitored automatically within the social context.

Endophenotypes that relate to depression can also be studied in semi-natural setups. Some evidence suggests that the individual response to SSRIs might be affected by socioeconomic status and self-esteem [62, 63]. Moreover, from an evolutionary perspective, some core symptoms of depression, such as social withdrawal and anhedonia, may have evolved to enhance survival by regulating the response to a conspecific threat. According to the Involuntary Defeat Strategy (IDS) theory [64], individuals can often avoid injury or death by accepting a new social status. However, suppose individuals cannot come to terms with their new social status. In that case, the stress response system remains highly activated. The continuous inner struggle for a potentially hopeless cause may eventually lead the individual to excessive submissiveness, poor self-esteem, social anxiety, and eventually depression. Such endophenotypes can be measured in ethologically relevant setups. In a semi-natural environment, prominent and stable social dominance hierarchies (SDH) between males emerge naturally, and thus, SDH and consequent social stress can be monitored and even manipulated. Tests for anhedonia, motivation, executive cognitive function, and exploration in a semi-natural setup are also feasible and allow monitoring of changes in appetite and the effects of drugs over prolonged periods.

## Ethologically relevant setups for rodents

### Home-Cage Monitoring

Home-Cage Monitoring (HCM) refers to the continuous, undisturbed, and automatic monitoring of rodents in their home cage. HCM can potentially minimize the ‘experimenter effect’- the compromised standardization and reproducibility in behavioral tests caused by the gender of the experimenter or by differences in handling styles [65]. Prolonged monitoring of behavior in the home cage also increases statistical power and enables the analysis of the temporal dynamics of behavior [66, 67]. However, individual caging of mice and rats affects their mental state and behavior. Thus, prolonged individual caging is not suitable for modeling mental disorders [68]. Prolonged monitoring of individually caged mice can be done with an array of infrared (IR) beam detectors and transmitters that surround the cage [69] or mechanosensory devices (piezo-electric sensors) that are placed below the cage [65]. However, HCM techniques for tracking the locomotion of grouped mice while keeping their identities are more complex. Radiofrequency identification (RFID) technology, for instance, is based on tags that are subcutaneously implanted to all group members and remain passive until an animal enters the electromagnetic field generated by the RFID antenna, which activates the tag to signal back a specific ID. The antennas can be localized in specific entrances/exits of predefined zones. For instance, the Intellicage system has been used to identify individuals as they enter the corners of the cage to perform a designed rewarding task. It is a relatively large cage, and it enables the design and application of different paradigms such as place preference, impulsivity, and working memory [70, 71]. However, social interactions and other behaviors that occur outside of the restricted zones are left undetected [65].

### Semi-natural setups- data acquisition by skilled observers

HCM provides continuous information on mice locations in a standard cage, nevertheless, a substantial body of evidence demonstrates the importance of an enriched and ethologically relevant environment to the display of elaborate, genuine, and translationally relevant behavioral repertoires [36]. Robert and Caroline Blanchard pioneered the semi-natural approach in the laboratory when they developed the Visible Burrow System (VBS), which contains artificial burrows, chambers with food and water, and an open zone that is exposed to a light/dark regime. The whole enclosure (1 m^2^ in size) is video recorded from above, and skilled observers watch the videos at specific time windows to monitor specific behaviors [72, 73]. Studies with the VBS showed that the strength of the SDH depends on the social environment. For instance, subordinate males lose weight in a colony of 6 males and 2 females but not in groups of just 3 males.

Moreover, subordinate mice constituted two group types - “stress-responsive” and “stress non-responsive”. Individuals in the non-responsive subtype failed to demonstrate a normal corticosterone elevation response to acute stress following 14 days in the VBS. They had a reduced corticotropin-releasing factor (CRF) mRNA in the amygdala compared to controls. Those individual differences in coping strategies may shed light on susceptibility versus resilience in developing mental illnesses in humans [74, 75]. Subordination also affects metabolism, with subordinates showing lower insulin and leptin levels while in the VBS and hyperinsulinemia and hyperleptinemia following the recovery phase outside of the VBS [76, 77]. Hence, correlates of SDH and other parameters of social status in mice in semi-natural setups with metabolic parameters can provide new insights into the metabolic syndrome [78]. Sequential analyses of the temporal dynamics of behavior can also be insightful. For instance, it has been shown that in the VBS, the percentage of frontal approaches that resulted in flight responses was significantly lower than the percentage of face-to-back approaches, suggesting that the latter is a more agonistic behavior [72].

How ‘natural’ should a ‘semi-natural’ setup be? There is a trade-off between the potential insights drawn from a highly nature-like environment and the difficulty of setting up such an environment and observing and analyzing the data. James Curley’s group established a complex vivarium, 150 × 80 cm in size, for mice to live in, which comprises of a ground-level that is made of interconnected nest boxes and an above-ground level that contains food, water, and other objects for enrichment. The setup is observed in real-time by trained observers, 2 h per day, for different behaviors such as flee, fight, chase, mount, and submission [79]. In one study, four such setups were interconnected, and 30 individual ICR mice were marked on their fur and introduced inside. Aggressive displays between the mice indicated that they were divided into two major sub-communities of 19 and eight individuals (with 3 animals not conclusively belonging to any of them). Each sub-group engaged in aggressive encounters at different locations of the four connected vivaria [79]. Using this complex setup, the authors concluded that activity levels measured in classical tests before group formation are not related to the eventual social network position, suggesting that these canonical tests are unreliable for predicting long-term social behaviors in groups. In another study, as determined by the Tube Test, aggression was not predictive of dominance in groups of 12 male mice later tested in the vivarium [80]. Interestingly the mice formed transitive (meaning if A beats B and B beats C then A beats C), stable and long-lasting hierarchies, which suggests that each individual could recognize its social status, as well as that of all other group members [80]. Using a cross-correlation method (Spike Time Tiling Coefficient) on observational data [81], the authors could examine how likely a specific behavior of an individual was to occur following (within a 2 s time window) a given behavior of another individual. They found that subordinates monitor and react to the behavior of higher-ranking individuals and thus exhibit a so-called “attention hierarchy” [82].

These relatively complex studies are essential for demonstrating the limitations of classical tests to fully reflect the complexity of the group social structure and the potential of ethologically oriented paradigms to complement them. However, setups such as the VBS, that are based on data acquisition by skilled observers have been used for quite a while [73], but the considerable effort it demands in operation, and in data collection prevented such setups from being more widely adopted by the scientific community. Hence, automatization of behavioral measuring is a primary condition for bringing semi-natural setups to the forefront.

### Computational ethology-automatic detection of pairs behavior

The emerging field of ‘computational ethology’ utilizes advances in machine learning (ML) and computer vision for automatically detecting and analyzing behavior in freely behaving animals [83–86]. By combining computational ethology and HCM, scientists can detect complex behaviors of single or pairs of rodents from video recordings. For instance, Hong et al. [87] tracked pairs of mice of different natural fur colors by combining videos from the side and top cameras under red light illumination and a depth-sensing camera. The authors extracted a low-dimensional representation of the mice (the pose estimation) and obtained manually annotated attacks, mountings, and social proximity. Next, they trained an ML classifier and demonstrated that it could reliably classify new events that were not used for training. Recently, the same group introduced the Mouse Action Recognition System -MARS [88], an automated pipeline of pose estimation and behavior quantification in pairs of freely behaving mice of different fur colors in a home cage. MARS can analyze videos taken from only a single top camera, simplifying the first step of data acquisition. The pose estimation data is further subjected to feature extraction – namely, numerous calculations of the relationship between body parts in each frame and between frames. These features are then fed into a classifier trained on 14 h of annotated behavioral data. MARS automatically detects attacks, mounts, social proximity and can potentially be trained to identify other complex behaviors between two mice during a short home cage interaction. Another tool for automatic detection of complex behavior in the home cage is named the ‘Alpha Tracker’ [89], which is capable of tracking pairs of mice of the same fur color. The above is but a fraction of the available tools for automatic behavioral detection.

The rodent’s flexible skeleton makes pose estimation, even if recorded in high-quality conditions, quite a challenge. An exciting breakthrough in video-based pose estimation of rodents was achieved by utilizing advanced ML techniques (supervised deep neural networks- DNN) [90–92]. DeepLabCut (DLC), for instance, is an accessible open-source tool for accurate pose estimation and tracking of experimenter-defined body parts in noisy and variable environments. DLC tackles the need for DNN to be trained on huge amounts of data using an approach called transfer learning, wherein, instead of training DNN “from scratch”, models previously trained to classify images are “fine-tuned”, significantly reducing the amount of labeled data needed to levels that most research groups can afford to manually label. The developers have demonstrated that with transfer learning, even a small number of training video frames (~200) from a novel setup (e.g., a behavioral test with mice) is enough to train the network to within human-level accuracy.

Of note, many of the computational ethology platforms are open source and designed with user-friendly interfaces. For instance, tools such as SimBA (SIMple Behavioral Analysis) [93] are often coupled with graphical user interfaces that do not require advanced computational skills and can help manage pose estimation data from DLC. Moreover, for specific tests such as the Resident Intruder Assay, the developers already created detailed definitions of behavioral readouts for mice and rats and demonstrated accurate automatic detection of these predefined behaviors.

With ML tools becoming more widely available, questions regarding proper and standardized annotation of behavior become critical. The best way to achieve consistency and reproducibility is by training a team of annotators to recognize well-defined behavioral events and establish large behavioral libraries. However, even skilled observers will typically agree with each other on only ~70–90% of the frames, depending on the specific behavior of interest and other technical issues, such as video quality, with disagreement mainly occurring at the beginning and end of events [93]. This raises an important question: what constitutes a meaningful unit of behavior? Since non-restricted behaviors are complex, occur at different time scales ranging from sub-seconds to minutes and even days, and may vary between different physical and social environments, often there is no clear answer to this question [94].

In light of that, researchers search for ways to quantify behavior without predefined labeling. Ethology assumes that behavior is modular – i.e., made up of repeated and stereotypical behavioral motifs. The brain strings these motifs into sequences so that the animal can execute appropriate and complex actions that reflect its internal state (e.g., time-varying needs and goals). An exciting subfield within computational ethology searches for such motifs – the ‘syllables of the behavioral language’ – by utilizing unsupervised ML approaches. The idea is to parse behavior into units of action, based upon the underlying statistical structure, without relying upon explicit human-supplied labels. This subfield of research, called computational neuro-ethology, further links such motifs and other types of behavioral representations with neuronal activity. For example, Motion Sequencing (MoSeq) is a tool for modeling and analyzing 3D depth-sensing behavioral data [95]. MoSeq uses computational modeling to automatically identify behavioral modules and sequences that best explain the patterns in any given experimental dataset. The modules can be regarded as the syllables of the behavioral language and the statistics that govern their associations over time as the behavioral grammar. Notably, the number and contents of the modules are not explicitly predefined - but instead inferred from the data. In agreement with classical ethology, Wiltschko et al. [95] found that mice exposed to the fox odor trimethylthiazoline exhibit fear-related behavior that is not composed of new syllables but rather of new sequences of the same syllables displayed during normal locomotor activity. In another study from the same group [96], the authors showed that the striatum might be involved in concatenating sub-second 3D behavioral motifs into action sequences during unrestricted behavior. Notably, the specific readouts that are fed into MoSeq, which are the depth-sensing data that represent the posture of a single mouse in a specific arena, together with the authors’ computational modeling choices, might affect the extracted syllables.

### Semi-natural setups- automatic detection of groups

Ethology aims to study animal behavior in their natural habitat. Computational ethology is inspired by ethology and aims to measure complex behavior in the laboratory in a natural context. Currently, computational ethology focuses mainly on short dyadic interactions in the animals’ home-cage. Nevertheless, a significant goal of computational ethology would be to measure behavior in semi-natural setups over long time periods [97]. An automatic semi-natural setup should enable the tracking of each individual within the group for as much time as the researcher requires, under a naturalistic light-dark cycle. The enriched 3D environment should include essential resources such as food and water, as well as potential mating partners, social competitors, and sheltered hiding places. The acquired data’s spatiotemporal resolution should be high enough to extract complex behaviors, such as chases, attacks, grooming, and mating.

Furthermore, the setup should enable standardized biological repeats (each repeat is one group), as well as remote brain recording and neuronal manipulation, food foraging, exposure to threats, and rewards. Also, technical simplicity, in terms of establishment and operation, and cost-effectiveness compared to classical behavioral setups is a crucial factor when it comes to adoption by the broad scientific community. Clearly, for specific questions, semi-natural setups that address only part of the requirements mentioned above can still be complimentary or advantageous over classical tests [98, 99]. For instance, in the Eco-Hab system, group-housed animals can live in a spacious four-compartment enriched apparatus bridged by tube-shaped corridors. RFID antennas are placed on both ends of each corridor. The time spent together in the same chamber with another mouse is automatically measured and taken to reflect its sociability.

Nevertheless, video-based techniques are required to detect complex behaviors such as fights or chases. Our group has developed a semi-natural setup named the ‘Social Box’ (SB) [100] based on video color recognition. The SB enables uninterrupted monitoring of individual and pairwise complex behaviors in a group (e.g., feeding, contacts, chases) for prolonged periods. We have also successfully incorporated technologies to manipulate and record brain function in groups of behaving mice in the SB. The system is easy to establish, affordable, and compatible with pose estimation methods such as DLC and multiple arenas that can be run in parallel for sufficient statistical power. The arenas (60 cm^2^) are enriched with bedding, feeders, water, nests, and other objects, including ramps and a sheltered hiding place. The mice are identified throughout the light and dark (2 lux illumination) phase of the day by color marking their fur with distinctive hair dyes that remain visible for a few weeks. A top single, color camera sensitive to dim light records videos 24/7. We can simultaneously collect videos from 12 such areas, containing mostly groups of 4 mice. In the first study using the SB [100], we automatically monitored the mice’s visits into the ten most frequently visited regions in the arena, based on the centroids of each individual’s body, and extracted the distribution of all the spatial configurations of the four mice in these regions. We then built a hierarchy of maximum entropy models to describe the group configurations based on successive orders of correlations between the mice (i.e., a model that assumes the location of each individual is independent of the others, and models that additionally capture pairwise, or third-order statistical dependencies). Models based solely on the behavior of individuals could not accurately describe the group-wise location configurations of the group members. This indicates that the mice are not independent entities in terms of spatiotemporal behavior. Surprisingly, neither could the models that focused on interactions between pairs of mice. Only models that included interactions between three mice gave a good approximation of the observed locations of the group. Hence, more than one group member influences a mouse’s location in the SB. We also found that animals that had lived in larger groups and enclosures that were more complex were influenced more by pairwise interactions and less by three-way interactions. These findings emphasize the relevance of the group context for social behavior and suggest that being raised in a complex environment strengthens mouse individuality.

The ability to study individuality in a semi-natural paradigm is of great translational significance. Individual differences in behavior originating from different personalities can be a major cause of individual responses to stressors, affecting resilience, susceptibility, and predisposition to mental illnesses [101]. We, therefore, developed a model that captures and outlines stable personality traits in mice [102]. Automatic location tracking of individuals allows for high throughput behavioral data collection, yielding a total of 60 behavioral readouts including both individual (e.g., locomotion, exploration, and foraging patterns) and social (e.g., approaches, contacts, and chases) behaviors measured across four days. We used linear discriminant analysis to reduce the dimensionality of this 60-dimensional behavioral space, inferring dimensions that maximize the ratio of inter-subject to intra-subject variability, i.e., dimensions that capture distinctive and stable individual behavioral information. The analysis yielded four prominent dimensions of identity domains (IDs). In addition, we found that the IDs were sufficient to predict multiple behavioral readouts across standard tests.

The ability to activate or inhibit specific neuronal populations in behaving animals using chemogenetics or optogenetics has revolutionized the field of neuroscience. We have utilized both techniques to investigate causal relationships between brain and behavior in the SB in two separate studies. In the first study [103], we found that during a short dyadic test, pharmacological delivery of Urocortin3 (Ucn3), a component of the CRF stress system, into the medial amygdala (MeA) caused mice to exhibit decreased and increased interaction through a mesh with a familiar and an unfamiliar conspecific, respectively. However, interaction through a mesh can be difficult to interpret since either prosocial or aggressive motivation can drive it. When we expressed hM4Di in the MeA of groups of Ucn3-Cre mice in the SB and inhibited the MeA-Ucn3 neurons by giving CNO in the drinking water, we observed an increase in the time the mice spent outside the nest and in the number of contacts between them. We interpreted these changes as an increase in prosocial behavior. Thus, by incorporating chemogenetics into the SB and measuring two relatively simple readouts, we could better understand the role of Ucn3 in social behavior. Optogenetics gives greater temporal control of neuronal activity than chemogenetics, but light that stimulates a specific brain target requires optic fibers and thus was previously unfeasible in the SB. In a more recent study [104], we developed a wireless optogenetic device and successfully activated oxytocin-expressing neurons in groups of mice for extended periods. Using this novel technique, we found that in a group of mice in the SB, oxytocin might promote prosocial and aggressive behaviors. This finding provides strong evidence that oxytocin regulates the salience of external social cues rather than strictly promoting affiliative behaviors. This is an important discovery, given that oxytocin is being promoted as a therapeutic avenue for ASD.

We also successfully incorporated into the SB miniature wireless electroencephalographic (EEG) recording devices [105]. This technology, ideal for recording brain-wide, synchronized, oscillatory neural activity inside the SB arenas, enables us to examine the influence of a rich social environment on such neural dynamics. Since disturbances in social functioning are comorbid with sleep problems, we recently used this technology to investigate the effect of the social environment on individuals’ sleep patterns, neural activity, and behavior. We found increased dark phase slow-wave activity combined with a pronounced increase in light phase REM sleep in subordinate mice, which may reflect a response to aggressive social interactions during the preceding dark phase. Indeed, SDH is a significant component of the social environment in mice. In another study from our laboratory [106], we showed that individuals’ social dominance rankings significantly predict behavioral differences following exposure to chronic stress. Importantly this association carried opposite consequences for males vs. females. The SB is ideal for testing long effects of pharmacological therapeutic agents on complex behavior. For instance, the antidepressant effects of ketamine remain long after its metabolism. In a recent study, Lopez et al. demonstrated long-duration antidepressant effects in mice group behavior in the SB. [107].

Other groups also developed approaches for automatic tracking of group of mice in semi-natural environment. Ohayon et al. [108] developed a system that learns unique bleached fur patterns on the mice and tracks them during dark and light cycles based on videos from IR camera. The authors found that the frequency of social interactions increased over days, and the mice established a stable SDH (based on ‘follow’ behavior). Weissbrod et al. [109] used an RFID antenna array located at the bottom of the arena and time-synchronized and fused with video recordings from an IR camera. This hybrid system can provide the position, speed, and orientation of uniquely identified socially interacting individuals. Based on the trajectories of each mouse, the authors measured walking, running, and SDH ranks based on chases between the group members. de Chaumont et al. [110] developed the Live Mouse Tracker (LMT) that is based on a combined RFID antenna array, depth-sensing camera, and machine vision approaches to estimate the animals’ locations in enriched and dynamically changing environments. LMT can also detect head-to-head or head-to-anogenital proximity between group members and other behaviors such as approach, escape, and chase. Etholoop [111] is yet another approach with a major emphasis on reconstructing the naturalistic 3D environment, which enables tracking of small animals in an area that ranges from 1 to 100 m^2^ . A 4 g collar is put around their neck consisting of miniature battery-powered IR LEDs with different wavelengths. Three high-speed IR cameras identify and localize each individual in the environment and send the data to another camera that follows the position of the tracked mouse. This enables, for example, on-the-fly operant conditioning of remote-controlled reward boxes. The authors used DLC to track several body parts of each mouse and found that combining real-time tracking with remotely controlled elements can rapidly shape specific behaviors such as rearing.

## Concluding remarks

Methods for automatically tracking individual body parts in groups of rodents freely interacting in semi-natural paradigms are becoming more accurate, widely available, and easily implemented. As such, automated methods can play a pivotal role in merging ethology and behaviorism, benefiting from the merits while compensating for the weaknesses of each, and ultimately contributing to overcoming the translational gap in biological psychiatry [97]. However, big data in the form of pose estimation trajectories of each group member, and post-processing of the data, will bring new challenges in the experimental design and execution, computational modeling, and statistical analysis. These challenges will demand a collaborative effort to establish conventions and common language to benefit the broad scientific community. The availability of big, standardized datasets will enable researchers to construct more elaborate and accurate models, using both supervised and unsupervised learning approaches to test specific hypotheses.

Importantly the ability to collect and classify a large amount of behavioral data does not imply that the strength of the semi-natural approach is by conducting hypothesis-free experiments only. It is vital to complement hypothesis-generating experiments with standard manipulations of complex environments that go beyond observational and correlational studies. It will also be crucial to develop techniques that will enable us to collect continuously physiological measurements, for instance, telemetry-based ECG. Indeed, previous studies have demonstrated that heart rate variability can be considered a potential stress marker [112]. Hence the detection of altered heart rate variability using a telemetry implant can be indicative of individual stress response and coping. To conclude, we believe that the evolving RDoC framework together with utilization of various rodent species models in a dynamic but controlled semi-natural setup could be the Rosetta Stone for revealing neuronal mechanisms of emotions in humans.
